# Metabolic Engineering of *Escherichia coli* for the Production of Hyaluronic Acid From Glucose and Galactose

**DOI:** 10.3389/fbioe.2019.00351

**Published:** 2019-11-21

**Authors:** Ji Eun Woo, Hyeon Jeong Seong, Sang Yup Lee, Yu-Sin Jang

**Affiliations:** ^1^Department of Agricultural Chemistry and Food Science Technology, Division of Applied Life Science (BK21 Plus Program), Institute of Agriculture & Life Science (IALS), Gyeongsang National University, Jinju, South Korea; ^2^Department of Chemical and Biomolecular Engineering (BK21 Plus Program), BioProcess Engineering Research Center, Center for Systems and Synthetic Biotechnology, Institute for the BioCentury, Korea Advanced Institute of Science and Technology (KAIST), Daejeon, South Korea

**Keywords:** hyaluronic acid, galactose, *Escherichia coli*, UDP-*N*-acetyl glucosamine, UDP-glucuronic acid

## Abstract

Hyaluronic acid is a glycosaminoglycan biopolymer widely present throughout connective and epithelial tissue, and has been of great interest for medical and cosmetic applications. In the microbial production of hyaluronic acid, it has not been established to utilize galactose enabling to be converted to UDP-glucuronic acid, which is a precursor for hyaluronic acid biosynthesis. In this study, we engineered *Escherichia coli* to produce hyaluronic acid from glucose and galactose. The galactose-utilizing Leloir pathway was activated by knocking out the *galR* and *galS* genes encoding the transcriptional repressors. Also, the *hasA* gene from *Streptococcus zooepidemicus* was introduced for the expression of hyaluronic acid synthase. The consumption rates of glucose and galactose were modulated by knockout of the *pfkA* and *zwf* genes, which encode 6-phosphofructokinase I and glucose-6-phosphate dehydrogenase, respectively. Furthermore, the precursor biosynthesis pathway for hyaluronic acid production was manipulated by separately overexpressing the gene clusters *galU-ugd* and *glmS-glmM-glmU*, which enable the production of UDP-glucuronic acid and UDP-*N*-acetyl-glucosamine, respectively. Batch culture of the final engineered strain produced 29.98 mg/L of hyaluronic acid from glucose and galactose. As a proof of concept, this study demonstrated the production of hyaluronic acid from glucose and galactose in the engineered *E. coli*.

## Introduction

Hyaluronic acid is a viscoelastic and hygroscopic glycosaminoglycan polymer comprising glucuronic acid and *N*-acetyl glucosamine (Jongsareejit et al., 2007; Yu and Stephanopoulos, 2008) and is found in skin and intercellular space of tissues of human (Kogan et al., 2007; Marcellin et al., 2014). Hyaluronic acid has been used for treating abnormal immune function, tumorigenesis and inflammation, and has applications in medical (ophthalmology, rheumatology, and dermatology) and cosmetic fields (Laurent et al., 1996; Mahoney et al., 2001; Mao et al., 2009; Park and Kim, 2017; Cho et al., 2018). In 2018, global market size for hyaluronic acid has been valued at 8.3 billion US dollars (Grand View Research, 2019).

Hyaluronic acid can be extracted from animal tissues, including rooster combs, human umbilical cord, and bovine synovial fluid and vitreous humor (Liu et al., 2011); however, extraction of hyaluronic acid from animal tissues might yield a complex proteoglycan that carries a risk of cross-species immunogenicity. Alternatively, hyaluronic acid can be produced by microbial fermentation using *Streptococcus* species. For a long time, industrial-scale production of hyaluronic acid has been performed using a pathogen *Streptococcus zooepidemicus* (Kim et al., 1996; Schiraldi et al., 2010), which produces a pathogenic factor together hyaluronic acid that contributes to forming the extracellular capsule (Kim et al., 2019). Because of this risk, there has been increasing interest in engineering of non-pathogenic microorganisms, including genus of *Escherichia, Bacillus, Lactococcus*, and *Agrobacterium*, for hyaluronic acid production (Widner et al., 2005; Chien and Lee, 2007; Mao and Chen, 2007; Yu and Stephanopoulos, 2008).

As a host for hyaluronic acid production, *E*. *coli* offers the benefits of well-defined metabolic pathways and engineering tools and the lack of pathogenicity. In 2007, *E*. *coli* strain HMS174(DE3)-pLysS was engineered for hyaluronic acid production by introduction of the *S*. *zooepidemicus hasA* gene encoding hyaluronic acid synthase with a rare-codon modification (Jongsareejit et al., 2007). Several research groups further engineered *E*. *coli* for enhanced production of hyaluronic acid from glucose by introducing another synthase and optimizing the formation of the precursors, UDP-glucuronic acid and UDP-*N*-acetyl glucosamine (Yu and Stephanopoulos, 2008; Yu et al., 2008; Mao et al., 2009; Lai et al., 2016). UDP-glucuronic acid is synthesized from glucose through the Embden-Meyerhof-Parnas (EMP) route.

Otherwise, UDP-glucuronic acid can also be synthesized from galactose through the Leloir pathway. The galactose-utilizing Leloir pathway had not previously been optimized for hyaluronic acid production in *E. coli*, though galactose enable to be converted to UDP-glucuronic acid easily rather than glucose. In this study, we engineered the galactose pathway in *E. coli* to enhance UDP-glucuronic acid biosynthesis for hyaluronic acid production in the co-fermentation of glucose and galactose. Furthermore, the biosynthesis pathways for UDP-glucuronic acid and UDP-*N*-acetyl glucosamine were enhanced by individually overexpressing the corresponding genes in the engineered *E. coli*. As a proof of concept, our results demonstrate that hyaluronic acid production can be enhanced by reinforcing UDP-glucuronic acid biosynthesis through the combination of two pathways for galactose utilization and UDP-glucuronic acid synthesis, in the co-fermentation of glucose and galactose using the engineered *E. coli* strain.

## Materials and Methods

### Strains and Culture Conditions

Strains used in this study are listed in Table 1. *Escherichia coli* K12 W3110 was employed as a host strain for hyaluronic acid production. Genomic DNA of *Streptococcus zooepidemicus* ATCC 35246 was purchased from Korean Culture Center of Microorganisms (KCCM, Korea), and used as a template to amplify the *hasA* gene encoding hyaluronic acid synthase. *E. coli* strains were cultured in Luria-Bertani (LB) medium containing 5 g yeast extract, 10 g peptone, and 10 g NaCl per liter. When necessary, 50 μg/mL ampicillin (Ap; Phyto Technology Laboratories, USA), 25 μg/mL kanamycin (Km; Phyto Technology Laboratories), and 17.5 μg/mL chloramphenicol (Cm; Phyto Technology Laboratories) were supplemented into the medium. Isopropyl β-D-1-thiogalactopyranoside (IPTG; Georgiachem, USA) was used at a final concentration of 1 mM for induction of gene expression when indicated.

**Table 1:** Strains, plasmids, and gDNA used in this study.

**Name**	**Genotype**	**References**
***E. coli*** **STRAINS**		
K12 W3110	Coli genetic stock center strain No.4474	CGSC
TOP10	*mcrA* Δ(*mrr*-*hsdRMS*-*mcrBC*) ϕ80*lac*ZΔM15 Δ*lacX*74 *recA*1 *araD*139 Δ(*ara*-*leu*)7697 *galU galK rpsL endA*1 *nupG*; cloning host	Thermo Fisher
HA01	*E. coli* K12 W3110, pTac15k-*hasA*	This study
HA02	*E. coli* K12 W3110, Δ*galR*, Δ*galS*, pTac15k-*hasA*	This study
HA03	*E. coli* K12 W3110, Δ*galR*, Δ*galS*, Δ*pfkA*, Δ*zwf*, pTac15k-*hasA*	This study
HA03GlcNAc	*E. coli* K12 W3110, Δ*galR*, Δ*galS*, Δ*pfkA*, Δ*zwf*, pTac15k-*hasA*, pTrc99A-*glmSU*-*glmM*	This study
HA03GlcA	*E. coli* K12 W3110, Δ*galR*, Δ*galS*, Δ*pfkA*, Δ*zwf*, pTac15k-*hasA*, pTrc99A-*galU*-*ugd*	This study
**PLASMIDS**		
pCW611	Ap^R^, λ-Red recombinase under arabinose-inducible *P_*BAD*_* promoter, Cre-recombinase under IPTG-inducible *lacUV5* promoter, temperature sensitive origin	Song and Lee, 2013
pMtrc9	*trc* promoter downstream of *lox66-cat-lox71* cassette	Kim et al., 2008
pTac15k	Km^R^, *tac* promoter, p15A ori	Lab stock
pTac15k-*hasA*	pTac15k containing the *hasA* from *S*. *zooepidemicus* ATCC 35246	This study
pTrc99A	Ap^R^, *trc* promoter, pBR322 origin	Pharmacia
pTrc99A-*galU*-*ugd*	pTrc99A containing the *galU* and *ugd* from *E*. *coli* K12 W3110	This study
pTrc99A-*glmSU*-*glmM*	pTrc99A containing the *glmS, glmM*, and *glmU* from *E*. *coli* K12 W3110	This study
**gDNA**		
*Streptococcus zooepidemicus* ATCC 35246	Wild type, HA^+^, Lac^+^, Em^S^	KCCM

For flask cultures, seed cultures were prepared in the 20-mL test tube containing 5 mL LB medium. One milliliter of seed culture was transferred into the 500-mL flask containing 100 mL of fresh LB medium. All *E. coli* strains were cultured at 37°C and 200 RPM in shaking incubator (IST-4075, Jeiotech, Korea).

### Construction of Knockout Mutants

In *E. coli*, the *galR, galS, zwf*, and *pfkA* genes were knocked-out by the modified one-step inactivation method (Song and Lee, 2013). First, the linear DNA fragments for gene knockout were prepared by two-step polymerase chain reaction (PCR; MiniAmp^TM^ Thermal Cycler, Thermo Fisher, Singapore), using template plasmid pMtrc9 (Kim et al., 2008; Nogrado et al., 2019) containing the *lox66-cat-lox71* cassette. For example, to construct the linear DNA fragments for knockout of the *galR* gene, primers galR-KO-F1, galR-KO-R1, galR-KO-F2, and gal-KO-R2 were used (Supplementary Table 1). In the first PCR with primers galR-KO-F1 and galR-KO-R1, using pMtrc9 as a template, the 50-bp homologous arm sequences of the target gene were franked into the PCR products. In the next PCR with primers galR-KO-F2 and galR-KO-R2, additional 50-bp homologous extension were generated. Thus, the resulting PCR fragments contained 100-bp homologous arm sequences matched to the upstream and downstream regions of the *galR* gene. The other PCR fragments for knockout of the *galS, zwf*, and *pfkA* genes were also prepared by the same methods using the corresponding primers (Supplementary Table 1).

Next, *E. coli* harboring plasmid pCW611 (Song and Lee, 2013) was prepared by transformation of the plasmid into the target strain. Then, *E. coli* competent cells harboring plasmid pCW611 were prepared. For the preparation of competent cells, a colony was inoculated into LB medium supplemented with Ap, and cultured for 12 h at 30°C. One milliliter of cell cultures was transferred into 100 mL LB medium supplemented with Ap and 10 mM of arabinose (Sigma, USA) for induction of λ-red recombinase, and incubated at 30°C. After that, the electro-competent cells were prepared by the previous methods (Sambrook et al., 1989).

In final, the linear PCR fragments containing 100-bp homologous arm for the *galR* gene knockout were transformed into the competent *E. coli* cells by electroporation (Sambrook et al., 1989). Cells were then incubated at 30°C for 1 h, and spread on LB agar plate containing Cm. Recombinants were screened by colony PCR using primers galR-KO-F2, and galR-KO-R2 (Supplementary Table 1). Other primers used in this study were listed in Supplementary Table 1. The confirmed colonies were cultured on LB agar plate containing Cm and Ap. For the pop-out of the *cat* gene from chromosome, then, the colony was suspended in 1 mL LB broth, and spreaded on LB agar plate containing Ap together with IPTG for induction of Cre recombinase. The recombinants generated by pop-out recombination were screened by colony PCR using primers galR-KO-F1, and galR-KO-R1 (Supplementary Table 1). Then, plasmid pCW611 was cured by incubation at 42°C in the final *E. coli* strains.

### Assembly of Gene and Gene-Clusters

The *galU*-*ugd* and *glmSU*-*glmM* gene clusters were individually assembled with plasmid pTrc99a by the Gibson assembly method (Gibson et al., 2009). The *hasA* gene also assembled with plasmid pTac15k under the same condition. Plasmids pTrc99a and pTac15k were digested with restriction enzymes *AvaI* and *PstI*, respectively (Jun et al., 2019). Restriction enzymes used in this study were purchased from Enzynomics (Korea). The *galU* and *ugd* genes were individually amplified by PCR using *E. coli* gDNA as a template by primer pairs galU-Gib-F/galU-Gib-R and ugd-Gib-F/ugd-Gib-R containing 30-bp homologous sequence matched to the terminal of adjacent fragments (Supplementary Table 1). The *glmSU* and *glmM* genes were also individually amplified by the same methods using primer pairs glmSU-Gib-F/glmSU-Gib-R and glmM-Gib-F/glmM-Gib-R (Supplementary Table 1). The *hasA* gene was amplified by PCR using *S*. *zooepidemicus* ATCC 35246 gDNA as a template by primers hasA-F and hasA-R containing the 30-bp homologous sequences matched to the both ends of the linear pTac15K restricted by *PstI* (Supplementary Table 1).

Fifteen microliter of assembly mixture was mixed with 5 μL of DNA solution containing the equimolar amounts of restricted plasmids and PCR fragments. The final reaction mixtures were incubated at 50°C for 60 min. From these reactions, the recombinant plasmids pTrc99a-*galU*-*ugd*, pTrc99a-*glmUS*-*glmM*, and pTac15k-*hasA* were constructed.

### Purification and Quantification of Hyaluronic Acid

Hyaluronic acid was purified according to the precious reports (Yu and Stephanopoulos, 2008; Mao et al., 2009). One milliliter of 0.1% sodium dodecyl sulfate (SDS) solution was mixed with 1 mL cell broth. Then the mixture was incubated at room temperature for 10 min, followed by centrifugation at 13,000 rpm (LaboGene 1524, LaboGene, Korea) at 4°C for 10 min. The supernatant was mixed with three volumes of ethanol, and incubated at 4°C for overnight. The mixture was centrifuged at 5,000 rpm for 20 min. In final, the pellet was dissolved in 1 mL of filtered 0.15 M NaCl solution.

Hyaluronic acid was quantified by a modified turbidimetric method using cetyl trimethylammonium bromide (CTAB) (Nicola, 1955; Chen and Wang, 2009). A five-hundred microliter of 0.2 M sodium acetate buffer was added into the prepared 500 μL purified hyaluronic acid samples, and incubated at 37°C for 10 min. Then, 1 mL CTAB, which was pre-wormed at 37°C, was added into the mixture. The optical density of the final solution was measured at 600 nm using a spectrophotometer (BioPhotometer® D30, Eppendorf, Germany).

### Determination of Molecular Weight of Hyaluronic Acid

To determine molecular weight of hyaluronic acid produced in this study, the purified samples were applied to a gel permeation chromatograph (EcoSEC HLC-8320 GPC, Tosoh, Japan) equipped with RI detector and 2X TSKgel GMPWxl + TSKgel G2500PWxl column (7.8 × 300 mm). The elution rate was controlled at 1.0 mL/min at 40°C. From the analysis, weight average molecular weight (Mw) and number average molecular weight (Mn) were determined.

### Analytical Methods

The cell growth was monitored by determining dry cell weight (DCW). To measure DCW, cells were harvested from 5 mL culture broth at 13,000 rpm for 5 min. After drying for 12 h, the cell weight was measured using a balance (PRACTUM124-1SKR, Sartorius, Germany). The resulting values were presented as gDCW per liter. Concentrations of glucose and galactose in broth were determined using high performance liquid chromatography (HPLC; 1515, Waters, USA) equipped with refractive index detector (2414, Waters) (Woo et al., 2018). A MetaCarb 87H column (Agilent, USA) was used with a mobile phase of 0.01 N H_2_SO_4_ at 25°C at a flow rate of 0.5 mL/min.

## Results

### Heterologous Expression of *S. zooepidemicus* Hyaluronic Acid Synthase in *E. coli*

**Figure 1:**
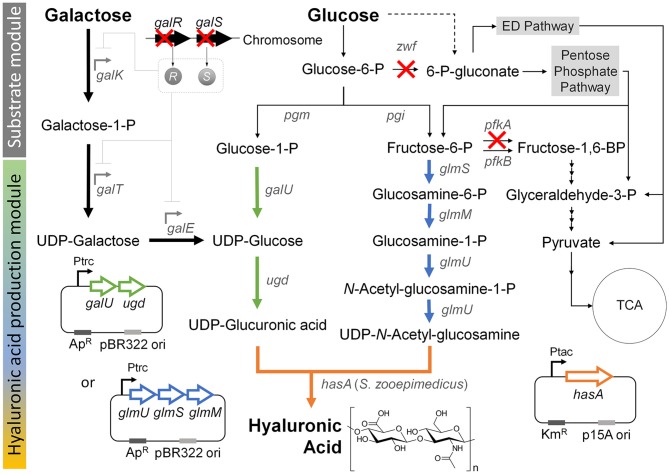
Metabolic engineering strategies for the production hyaluronic acid from the simultaneous consumption of glucose and galactose in *E*. *coli*. “X” indicates gene knockout. Thick black arrow indicates Leloir pathway, which was activated by knockout of the *galR* and *galS* genes in order to enhance the galactose consumption in the co-fermentation with glucose. Thick green arrow indicates the UDP-glucuronic acid biosynthesis pathway, which was reinforced by overexpression of the *galU* and *udg* genes under control of the *trc* promoter. Thick blue arrow indicates the UDP-*N*-acetyl glucosamine biosynthesis pathway, which was reinforced by overexpression of the *glmU, glmS*, and *glmM* genes under control of the *trc* promoter. Thick orange arrow indicates the heterologous expression of the *hasA* gene from *S. zooepidemicus*. Dashed line indicates unannotated pathway in glycogen metabolism (Long et al., 2016). Gene and its coding enzyme: *galR*, DNA-binding transcriptional repressor; *galS*, DNA-binding transcriptional isorepressor; *galK*, galactokinase; *galT*, galactose-1-phosphate uridylyltransferase; *galE*, UDP-galactose-4-epimerase; *pgm*, phosphoglucomutase; *pgi*, glucosephosphate isomerase; *zwf*, glucose-6-phosphate dehydrogenase; *galU*, glucose-1-phosphate uridylytransferase; *ugd*, UDP-glucose 6-dehydrogenase; *glmS*, L-glutamine:D-fructose-6-phosphate aminotransferase; *glmM*, phosphoglucosamine mutase; *glmU*, glucosamine-1-phosphate *N*-acetyltransferase; *pfkA*, 6-phosphofructokinase I; *pfkB*, 6-phosphofructokinase II; and *hasA*, hyaluronic acid synthase from *S. zooepidemicus* ATCC 35246.

Previous studies showed that hyaluronic acid could be formed by introducing the *S. zooepidemicus hasA* gene, which encodes hyaluronic acid synthase, into *E. coli* (Jongsareejit et al., 2007; Yu and Stephanopoulos, 2008; Yu et al., 2008; Lai et al., 2016). This enabled production of the hyaluronic acid from the precursors, UDP-glucuronic acid and UDP-*N*-acetyl glucosamine, through the native metabolic pathway in wild-type *E. coli* (Figure 1). Here, to construct a new basal strain for hyaluronic acid production, we expressed the *hasA* gene from *Streptococcus zooepidemicus* ATCC 35246 under the control of the *tac* promoter on plasmid pTac15k in *E*. *coli* K12 W3110. The obtained strain was named HA01.

**Figure 2:**
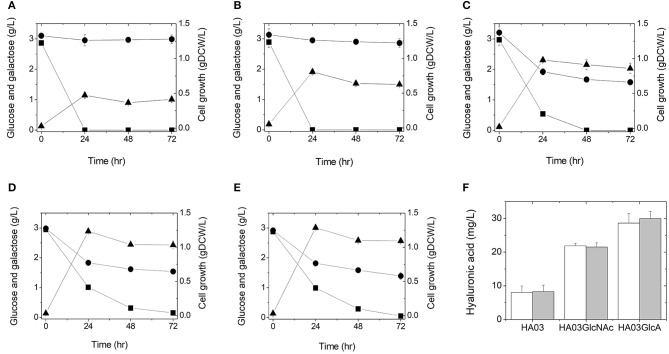
Culture profiles **(A–E)** and hyaluronic acid productions **(F)** of the engineered *E. coli* strains in a 500-mL flask containing 100 mL LB medium supplemented with 3 g/L glucose and 3 g/L galactose: HA01 **(A)**, HA02 **(B)**, HA03 **(C)**, HA03GlcNAc **(D)**, and HA03GlcA **(E)**. Symbols are: glucose (■), galactose (●), and gDCW (▲). Cultures were independently performed in triplicate. **(F)** Hyaluronic acid titers were determined from the triplicate cultures at 24 h (white) and 48 h (gray).

To test whether hyaluronic acid could be produced from glucose in this manner, we cultured *E. coli* strain HA01 in LB medium supplemented with 3 g/L glucose (3 g/L galactose was also added to keep consistency for all cultures, although HA01 strain cannot use galactose under both sugars condition). However, no production of hyaluronic acid was observed and only glucose was consumed (not galactose; Figure 2A). The observed cell growth was 0.47 gDCW/L at 24 h of culture (Figure 2A and Table 2).

**Table 2:** Batch culture results obtained from the metabolically engineered *E. coli* strains in this study.

**Strains**	**Glucose consumption rate (R_**Glc**_; g/L/h)**	**Galactose consumption rate (R_**Gal**_; g/L/h)**	**R_**Gal**_/R_**Glc**_**	**Cell mass^**a**^ (gDCW/L)**	**Specific growth rate (/h)**	**Hyaluronic acid titer (mg/L)**	**Hyaluronic acid productivity (mg/L/h)**
HA01	0.1195 ± 0.0012	0.0015 ± 0.0021	0.01	0.47 ± 0.04	0.0366 ± 0.0017	N.D.^b^	N.D.
HA02	0.1206 ± 0.0076	0.0038 ± 0.0042	0.03	0.81 ± 0.04	0.0350 ± 0.0053	N.D.	N.D.
HA03	0.0620 ± 0.0037	0.0226 ± 0.0029	0.36	0.98 ± 0.03	0.0530 ± 0.0116	8.30 ± 1.91	0.1729 ± 0.0398
HA03GlcNAc	0.0393 ± 0.0003	0.0212 ± 0.0018	0.54	1.23 ± 0.03	0.0419 ± 0.0040	21.52 ± 2.12	0.4483 ± 0.0272
HA03GlcA	0.0388 ± 0.0006	0.0200 ± 0.0002	0.52	1.29 ± 0.04	0.0462 ± 0.0138	29.98 ± 1.30	0.6246 ± 0.0442

a *Maximum cell mass (gDCW/L)*.

b *N.D., not detected*.

### Weak Galactose Uptake by Knocking-Out *galR* and *galS* Is Not Enough to Produce Hyaluronic Acid

Although *E. coli* strain HA01 showed no production of hyaluronic acid, the proper expression of hyaluronic acid synthase was observed, as assessed by SDS-PAGE (data not shown). We suspected that the precursors were inefficiently supplied for the production of hyaluronic acid by this strain.

We speculated that pairing galactose utilization with glucose consumption could produce UDP-glucose to increase the UDP-glucuronic acid pool for hyaluronic acid production. The action of UDP-glucose 6-dehydrogenase (encoded by the *ugd* gene) can directly from the hyaluronic acid precursor, UDP-glucuronic acid, directly from UDP-glucose, which is the final metabolite of the galactose-utilizing Leloir pathway (Figure 1). In an effort to increase the UDP-glucuronic acid pool by galactose consumption, we simultaneously deleted the *galR* and *galS* genes (Lim et al., 2013), which encode a DNA-binding transcriptional repressor and an isorepressor in the carbon catabolite repression mechanism, from the chromosome of *E. coli* strain HA01. The resulting strain was designated HA02. In *E. coli*, the GalR repressor can bind to two operator sites, *O*_E_ and *O*_I_, and dimerize to loop DNA and inhibit transcription of the galactose operon (Golding et al., 1991; Weickert and Adhya, 1993). The GalS isorepressor is 85% homologous to GalR at the amino acid sequence level (Adhya and Shapiro, 1969; Adhya and Miller, 1979; Weickert and Adhya, 1992; Adhya, 1996; Møller et al., 2002).

To see if simultaneous disruption of the *galR* and *galS* genes would increase galactose consumption, we cultured HA02 cells in LB medium supplemented with 3 g/L galactose and 3 g/L glucose. Galactose consumption was detected at a rate of 0.0038 g/L/h, which was relatively slow compared to the glucose uptake rate of 0.1206 g/L/h (Figure 2B and Table 2). Hyaluronic acid production was not detected in the culture broth. HA02 cultures attained a cell mass of 0.81 g/L at 24 h of culture, which was 1.72 greater than that obtained from HA01 cells (Table 2). This suggests that the simultaneous deletion of the *galR* and *galS* genes resulted in the slight consumption of galactose with simultaneous glucose uptake in *E*. *coli*, but that this change did not supply sufficient precursors to enable hyaluronic acid production.

### Enhanced Galactose Consumption and Reduced Glucose Uptake Following Knockout of the Key Genes in the EMP and Pentose Phosphate (PP) Pathways

Given that activation of the Leloir pathway by knockout of the *galR* and *galS* genes enabled weak galactose consumption under co-fermentation with glucose, we next sought to modulate the pathway for glucose catabolism for enhancing galactose consumption. In wild-type *E. coli*, glucose is mainly catabolized through the EMP and PP pathways, which are responsible for ~88 and 11%, respectively, of this process (Nakahigashi et al., 2009; Hollinshead et al., 2016). To reduce glucose consumption through EMP and PP pathways, we herein knocked out the *pfkA* and *zwf* genes, which encode 6-phosphofructokinase I and glucose-6-phosphate dehydrogenase, respectively, in *E. coli* strain HA02 (Figure 1). The resulting strain was designated HA03. The enzymes encoded by *pfkA* and *zwf* are involved in the reactions that convert fructose-6-phosphate to fructose-1,6-bisphosphate in the EMP pathway and glucose-6-phosphate to gluconoiactone-6-phosphate in the PP route, respectively (Figure 1).

To test whether glucose catabolism would be limited in this strain during co-fermentation with galactose, *E. coli* HA03 cells were cultured in LB medium containing 3 g/L galactose and 3 g/L glucose. As expected, we observed a lower glucose uptake rate (0.0620 g/L/h) than that seen for HA02 cells, along with galactose consumption at a rate of 0.0226 g/L/h (Figure 2C and Table 2). In the co-fermentation of glucose and galactose, the hyaluronic acid production was 8.30 mg/L, which is 105 and 162% of the yields obtained in the glucose- and galactose- fermentations, respectively (Figure 2F and Table 2). These results indicate that knockout of *pfkA* and *zwf* and disruption of *galR* and *galS* led to the generation of a meaningful pool of UDP-glucuronic acid for the production of hyaluronic acid from galactose *via* UDP-glucose.

### Reinforcing the Biosynthesis of UDP-*N*-Acetyl Glucosamine

Next, we considered two additional options for enhancing hyaluronic acid production: 1) reinforcing the biosynthesis of the second relevant precursor, UDP-*N*-acetyl glucosamine; and 2) further enhancing the UDP-glucuronic acid pool (Figure 1). We examined the first option by overexpressing the *glmS, glmM*, and *glmU* genes encoding L-glutamine:D-fructose-6-phosphate aminotransferase, phosphoglucosamine mutase, and glucosamine-1-phosphate *N*-acetyltransferase, respectively, in strain HA03. The resulting strain was designated HA03GlcNAc. In *E. coli* HA03GlcNAc, additional transcription of the *glmS, glmM*, and *glmU* genes was controlled by the *trc* promoter on plasmid pTrc99a-*glmSU*-*glmM*. The enzymes encoded by the three genes were projected to facilitate the reaction cascade that yields UDP-*N*-acetyl glucosamine from fructose-6-phosphate (Figure 1).

To examine whether this reinforcement of UDP-*N*-acetyl glucosamine biosynthesis would increase hyaluronic production in the *galR*-*galS-pfkA*-*zwf* mutant *E. coli*, this strain was cultured in LB medium containing 3 g/L galactose and 3 g/L glucose. The culture of HA03GlcNAc produced hyaluronic acid at 21.52 mg/L, which was 2.59 times the value obtained from the parental HA03 strain (Figure 2F and Table 2). The production of cell mass was 1.23 gDCW/L in HA03GlcNAc cultures (Figure 2D and Table 2). The galactose uptake rate did not significantly differ between HA03 and HA03GlcNAc, while the glucose consumption rate of HA03GlcNAc was rather slow compared to that of HA03 (Table 2).

### Reinforcement of UDP-Glucuronic Acid Biosynthesis by Overexpression of the *galU* and *ugd* Genes

The second option for enhancing hyaluronic acid production was examined by overexpressing the *galU* and *ugd* genes encoding glucose-1-phosphate uridylyltransferase and UDP-glucose 6-dehydrogenase, respectively, in strain HA03. The resulting strain was designated HA03GlcA. Glucose-1-phosphate uridylyltransferase, which is encoded by the *galU* gene, is involved in converting glucose-1-phosphate to UDP-glucose, which could also be synthesized through Leloir pathway from galactose in strain HA03GlcA (Figure 1). The UDP-glucose pool generated by the two different routes could be converted to UDP-glucuronic acid by the *ugd* gene-encoded enzyme (Figure 1).

To examine whether overexpression of the *galU* and *ugd* genes and the expected increase in UDP-glucuronic acid biosynthesis would enhance hyaluronic acid production, we cultured HA03GlcA cells in LB medium containing 3 g/L galactose and 3 g/L glucose. Indeed, strain HA03GlcA produced 29.98 mg/L hyaluronic acid, and thus exhibited a production level that was 3.61 and 1.39 times those obtained using strains HA03 and HA03GlcNAc, respectively (Figure 2F and Table 2). The cell mass production for HA03ClcA was 1.29 gDCW/L (Figure 2E and Table 2). The molecular weight of the hyaluronic acid produced in the culture of *E. coli* strain HA03GlcA was measured by GPC. The weight-average molecular weight (Mw) value was determined to be 1,386.5 Da, yielding a polydispersity (Mw/Mn) of 2.84.

## Discussion

In this study, we demonstrate the production of hyaluronic acid from the simultaneous consumption of glucose and galactose in cultures of engineered *E. coli* capable of enhancing the biosynthesis of UDP-glucuronic acid. With the initial non-genome-edited *E. coli* strain, HA01, we did not observe the consumption of galactose upon co-fermentation with glucose. To enable galactose to be efficiently consumed when co-fermented with glucose, we used a one-step knockout method to delete the *galR* and *galS* genes, according to the previous report (Lim et al., 2013). A small degree of galactose consumption was observed in the *galR-galS-*mutant (strain HA02), but there was still no detectable production of hyaluronic acid. Hyaluronic acid production was observed in cultures of HA03, in which the *pfkA* and *zwf* genes were disrupted along with the knockout of *galR* and *galS*. The co-fermentation of galactose with glucose by the *galR-galS-pfkA-zwf-*mutant strain, HA03, yielded hyaluronic acid production of 8.30 mg/L. This result indicates that enhanced galactose consumption and reduced glucose uptake are important for hyaluronic acid production in *E. coil*. Using strain HA03, we further examined hyaluronic acid production following the overexpression of the genes that supply the precursors, *N*-acetyl glucosamine (*glmS, glmM*, and *glmU*) and UDP-glucuronic acid (*galU* and *ugd*). Interestingly, the overexpression of these gene sets in strain HA03 increased its hyaluronic acid production by 2.59 and 3.61 times, respectively, compared to the parental strain.

This is the first study wherein disruption of the *pfkA* and *zwf* genes and deregulation of carbon catabolite repression (CCR) through knockout of the *galR* and *galS* genes was used to enable the simultaneous and efficient utilization of glucose and galactose in *E. coli*. The *galR-galS-pfkA-zwf-*mutant consumed glucose and galactose at 0.0226 g/L/h and 0.0620 g/L/h, respectively, in batch culture using both carbon sources (Figure 2C and Table 2). The glucose consumption rate of the *galR-galS-pfkA-zwf-*mutant was rather slow compared to the 0.1206 g/L/h seen for the parent strain (Table 2). This reflects that the efficient uptake of galactose occurred *via* the decreased glucose consumption engineered by disruption of the *pfkA* and *zwf* genes, which decreased glucose catabolism through the EMP and PP pathways. In previous studies, carbon metabolism was redistributed from the EMP route toward the Entner Doudoroff (ED) and PP pathways by individual knockout of *pfkA* and *pgi* (encoding glucose-6-phaphate isomerase) in *E. coli* (Toya et al., 2010; Hollinshead et al., 2016; Seol et al., 2016). In cultures of the *pfkA* mutant grown using glucose as a sole carbon source, carbon metabolism showed a distribution of 24, 65, and 14% to the EPM, PP, and ED pathways, respectively, whereas the corresponding values in wild-type cultures were 88, 11, and 1%, respectively (Hollinshead et al., 2016). Taken together, the present findings suggest that glucose and galactose might be mainly catabolized *via* the ED pathway rather than the EMP and PP routes in the *galR-galS-pfkA-zwf-*mutant.

We also enhanced the production of hyaluronic acid in *E. coli* by employing the native *S. zooepidemicus hasA* gene and reinforcing the pathway for biosynthesis of UDP-glucuronic acid, as a proof of concept. The UDP-glucuronic acid biosynthesis pathway was reinforced by two different strategies: activation of the galactose pathway and overexpression of the pathway components. The strain harboring both alterations produced hyaluronic acid at 29.98 mg/L, which is much less than what was obtained in the previous report (Jongsareejit et al., 2007; Yu and Stephanopoulos, 2008; Yu et al., 2008; Mao et al., 2009; Schiraldi et al., 2010; Liu et al., 2011; Lai et al., 2016). Combination of our strategy together with the strategies reported in the previous works will give synergistic effect on the hyaluronic acid production. For example, one previous study used codon-optimized *S. zooepidemicus hasA*, while another co-expressed the *hasA* gene with *hasE* from the same source (Jongsareejit et al., 2007; Lai et al., 2016). In both cases, the genes encoding hyaluronic acid synthase were expressed under the control of the strong T7 RNA polymerase. The engineered strains produced hyaluronic acid at 32.5 and 127 mg/L, respectively. Another two research groups used the *hasA* gene from either *Streptococcus equisimilis* or *Pasteurella multocida* in *E. coli* (Yu et al., 2008; Mao et al., 2009). In the engineered *E. coli* expressing *S*. *equisimilis hasA*, the *ugd, galF* (encoding glucose-1-phosphate uridylyltransferase), and sigma factor *rpoS* genes were overexpressed, which resulted in hyaluronic acid production of 695.6 mg/L. In the engineered *E*. *coli* harboring the *hasA* from *P. multocida*, the *kfiD* gene encoding UDP-glucose-6-dehydrogenase was overexpressed (Mao et al., 2009). When grown with the precursor, glucosamine, and a cell-wall synthesis inhibitor, this strain produced 3.7 g/L hyaluronic acid.

We here in report the production of hyaluronic acid from an *E*. *coli* strain that we engineered for the simultaneous consumption of glucose and galactose. We first activated the Leloir pathway by eliminating two galactose operon repressors to create the *galR*-*galS*-mutant, which had glucose and galactose uptake rates of 0.1206 and 0.0038 g/L/h, respectively. In the second step, we controlled the pathway for glucose catabolism in the *galR*-*galS*-*pfkA*-*zwf* mutant; this reduced the glucose uptake rate (0.0620 g/L/h) and increased the galactose uptake rate to 0.0226 g/L/h. In the third step, we reinforced the UDP-glucuronic acid production pathway by constructing the *galR*-*galS*-*pfkA*-*zwf*-mutant harboring pTrc99A-*galU*-*ugd*; the glucose and galactose uptake rates were 0.0388 and 0.0200 g/L/h, and the strain produced hyaluronic acid at 29.98 mg/L. The strategy reported in this study will be helpful for constructing microbial strains capable of producing biochemicals from glucose and galactose.

## Data Availability Statement

The raw data supporting the conclusions of this manuscript will be made available by the authors, without undue reservation, to any qualified researcher.

## Author Contributions

Y-SJ, JW, and SL conceived the project. JW and HS performed experiments. JW, HS, and Y-SJ were involved in analysis and interpretation of experimental data. JW and Y-SJ wrote the manuscript. SL revised the manuscript. All authors read and approved the final manuscript.

### Conflict of Interest

The authors declare that the research was conducted in the absence of any commercial or financial relationships that could be construed as a potential conflict of interest.
