# Supplementary material for: Metabolic Engineering of Escherichia coli for the Production of Hyaluronic Acid From Glucose and Galactose
Source: Front Bioeng Biotechnol. 2019 Nov 21;7:351. doi: 10.3389/fbioe.2019.00351 (PMC6881274; doi:10.3389/fbioe.2019.00351)
Supplement: Supplementary file 1 [file Table_1.DOCX]

**Supplementary Material**

**Supplementary Table 1** Oligonucleotides Used in This Study.

| Name | Sequences (5’ → 3’) | Reference |
| --- | --- | --- |
| **Oligonucleotides** |  |  |
| galR-KO-F1 | ATGTAAGCGTTTACCCACTAAGGTATTTTCGACACTATAGAACGCGGCCG | This study |
| galR-KO-R1 | TTACTGGCGCTGGAATTGCTTTAACTGCGGCCGCATAGGCCACTAGTGGA | This study |
| galR-KO-F2 | AAAACACGCCACCCCTTGAACCAACGGGCGTTTTCCGTAACACTGAAAGAATGTAAGCGTTTACCCACTA | This study |
| galR-KO-R2 | TTGATAATGGTCAGGCGCGGTTGATTCGCCGTCGCCAGACCATCGAAGAATTACTGGCGCTGGAATTGCT | This study |
| galS-KO-F1 | ACTGTGAAATCACTCACAGATTGAAAGCGGGACACTATAGAACGCGGCCG | This study |
| galS-KO-R1 | CAGTCATTTACTGCAATCTCATAACAGGTACCGCATAGGCCACTAGTGGA | This study |
| galS-KO-F2 | CGGGTTACAACGTTAAAACGGTGCAATCATAGCTATCACATTGTTAAGATACTGTGAAATCACTCACAGA | This study |
| galS-KO-R2 | TCGATTCACGAAGTCCTGTATTCAGTGCTGACAAAATAGCCGCCAGCAAGCAGTCATTTACTGCAATCTC | This study |
| zwf-KO-F1 | TAAAATAACCATAAAGGATAAGCGCAGATAGACACTATAGAACGCGGCCG | This study |
| zwf-KO-R1 | TACCGGGTTAGTTAACTTAAGGAGAATGACCCGCATAGGCCACTAGTGGA | This study |
| zwf-KO-F2 | CCTGAAAGTGTAAAAATTGTTCTACAATCTGCGCAAGATCATGTTACCGGTAAAATAACCATAAAGGATA | This study |
| zwf-KO-R2 | AAAGCAGTACAGTGCACCGTAAGAAAATTACAAGTATACCCTGGCTTAAGTACCGGGTTAGTTAACTTAA | This study |
| pfkA-KO-F1 | GGTAAAGGAATCTGCCTTTTTCCGAAATCAGACACTATAGAACGCGGCCG | This study |
| pfkA-KO-R1 | TACTATTTGCACATTCGTTGGATCACTTCGCCGCATAGGCCACTAGTGGA | This study |
| pfkA-KO-F2 | CCTGATAAGCGAAGCGCATCAGGCATTTTTGCTTCTGTCATCGGTTTCAGGGTAAAGGAATCTGCCTTTT | This study |
| pfkA-KO-R2 | ATACCGCCATTTGGCCTGACCTGAATCAATTCAGCAGGAAGTGATTGTTATACTATTTGCACATTCGTTG | This study |
| hasA-F | CAGACCATGGAATTCGAGCTCGGTACCCGGATGAGAACATTAAAAAACCTCATAACTGTT | This study |
| hasA-R | CGCCAAAACAGCCAAGCTTGCATGCCTGCATTATAATAATTTTTTACGTGTTCCCCAGT | This study |
| galU-Gib-F | AATTGTGAGCGGATAACAATT ATGGCTGCCATTAATACGAAAGT | This study |
| galU-Gib-R | TCCGCCAAAACAGCCAAGCTTGCATGCCTGTTACTTCTTAATGCCCATCTCTTC | This study |
| ugd-Gib-F | AACAGACCATGGAATTCGAGCTCGGTACCCATGAAAATCACCATTTCCGGTACT | This study |
| ugd-Gib-R | GGCAGCCATAATTGTTATCCGCTCACAATTTTAGTCGCTGCCAAAGAGATC | This study |
| glmSU-Gib-F | AACAGACCATGGAATTCGAGCTCGGTACCCATGTTGAATAATGCTATGAGCGTAG | This study |
| glmSU-Gib-R | CATTATACGAGCCGGATGATTAATTGTCAATTACTCAACCGTAACCGATTTTG | This study |
| glmM-Gib-F | TTGACAATTAATCATCCGGCTCGTATAATGTGTGGAATTGTGAGCGGATAACAATTATGAGTAATCGTAAATATTTCGGTAC | This study |
| glmM-Gib-R | TCCGCCAAAACAGCCAAGCTTGCATGCCTGTTAAACGGCTTTTACTGCATCG | This study |
